# A ring-polymer model shows how macromolecular crowding controls chromosome-arm organization in *Escherichia coli*

**DOI:** 10.1038/s41598-017-10421-y

**Published:** 2017-09-19

**Authors:** Chanil Jeon, Youngkyun Jung, Bae-Yeun Ha

**Affiliations:** 1 Department of Physics and Astronomy, University of Waterloo, Waterloo, Ontario, N2L 3G1 Canada; 2 Supercomputing Center, Korea Institute of Science and Technology Information, Daejeon, 34141 Korea; 3 Present Address: School of Computational Sciences, Korea Institute for Advanced Study, Seoul, 02455 Korea

## Abstract

Macromolecular crowding influences various cellular processes such as macromolecular association and transcription, and is a key determinant of chromosome organization in bacteria. The entropy of crowders favors compaction of long chain molecules such as chromosomes. To what extent is the circular bacterial chromosome, often viewed as consisting of “two arms”, organized entropically by crowding? Using computer simulations, we examine how a ring polymer is organized in a crowded and cylindrically-confined space, as a coarse-grained bacterial chromosome. Our results suggest that in a wide parameter range of biological relevance crowding is essential for separating the two arms in the way observed with *Escherichia coli* chromosomes at fast-growth rates, in addition to maintaining the chromosome in an organized collapsed state. Under different conditions, however, the ring polymer is centrally condensed or adsorbed onto the cylindrical wall with the two arms laterally collapsed onto each other. We discuss the relevance of our results to chromosome-membrane interactions.

## Introduction

There has been a growing appreciation of entropic effects in organizing chain molecules^1–10^. This is most obvious for chromosome organization in bacterial cells, which lack internal partitioning^3–8^. The bacterial chromosome is not physically isolated from other macromolecules but still occupies a sub-cellular space known as the ‘nucleoid’^11^. Recent experiments suggest that *Escherichia coli* (*E. coli*) chromosomes behave as a ‘soft spring’ in a cylindrical space and can be entropically condensed in the presence of polymeric crowders^8^; the entropy of crowders favors chain collapse^4–10^. Indeed, macromolecular crowding has been the focus of several studies^1–3,7–10,12–14^. A key concept is the entropic (depletion) force between chain segments or monomers induced by crowding^15,16^. It is not only responsible for overall compaction of bacterial chromosomes but also governs their local organization by controlling the tendency of internal looping, as desired for such processes as transcription^3,8–10^.

**Figure 1:**
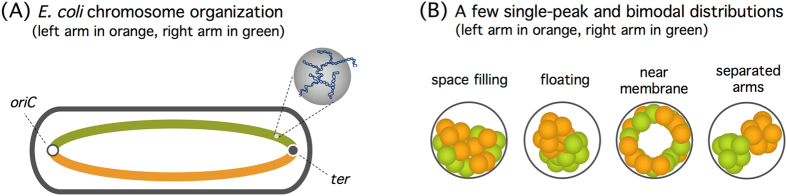
(**A**) Schematics of the *E. coli* chromosome consisting of two arms (left in orange and right in green). The chromosome resembles a ‘donut’ in fast growing cells, as illustrated in the figure, but it is asymmetrically organized in slowly-growing cells^5,20,21^. Also shown are *oriC* and *ter*, where replication is initiated and terminated, respectively. For simplicity, the topological complexity arising from DNA replication^17^ is not shown. (**B**) A few radial distributions of projected DNA segments; each sphere represents a supercoiled topological domain^6,8^ as shown in (**A**). (This figure is inspired by ref.^17^.) The emergence of two peaks is unique to the near-membrane and separated-arm distributions^17^. Note that the two arms (orange and green) are intermingled in the former but segregated in the latter.

Chromosome organization in elongated bacteria (e.g., *E. coli*) is of particular interest, because of anisotropic confinement the chromosome experiences. Accordingly, one has to characterize it in two different directions: longitudinal (along the long symmetry axis of the cell) and transverse (along the radial cell axis)^17,18^. Furthermore, a number of studies suggest that chromosome organization in *E. coli* cells is growth-rate dependent^3,8,17,18^. At fast growth rates, the (circular) *E. coli* chromosome is more or less symmetrically organized, resembling a donut; it consists of the two “arms” arranged in parallel (Fig. 1(A)) and is topologically more complex because of multi-fork replication^17^. At slow growth rates, there is evidence that it is asymmetrically organized, often cartooned as a ‘sausage’ with a stretch connecting the two ends^19–21^. [This is, however, not a universal feature of bacterial chromosomes but appears to be specific to *E. coli* (see a recent review^18^ for chromosome organization in other cells).]

**Figure 2:**
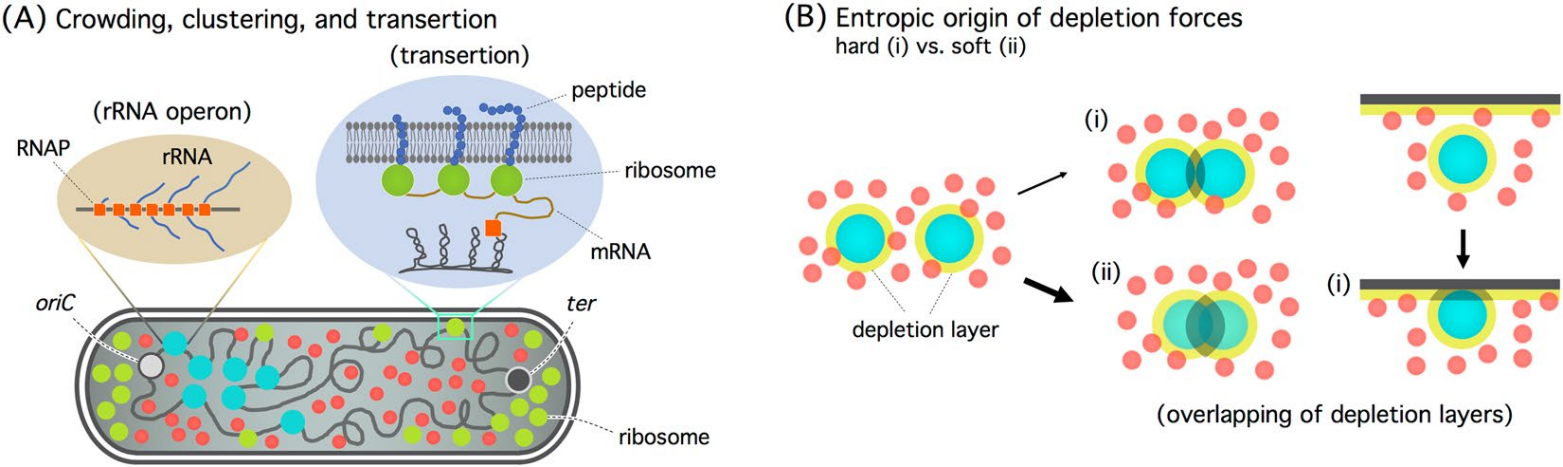
Clustering and transertion (**A**), and depletion forces (**B**). (**A**) The *E. coli* chromosome is a heterogeneous structure. In a polymer model, it can be viewed as consisting of big (cyan) and small (grey) monomers, with a tunable interaction with the inner cell membrane. The big monomer represents transcription-active sites in each rRNA (ribosomal RNA) operon decorated with RNAPs (RNA polymerases), each about 10 nm in size. The model illustrates a hypothetical *single* chromosome, which leaves out topological complexities arising from DNA replication. At fast growth rates, each big monomer contains many (about 70) RNAPs as well as RNAs they are making. What is also shown is the association of chromosome loci with the inner cell membrane through the insertion of membrane proteins in the membrane or simply ‘transertion’. This effect can be coarse-grained into a parameter describing the interaction of monomers with the confining cylindrical wall. (**B**) In a crowded medium, a large molecule (a sphere in cyan) can be considered as being surrounded by a depletion layer (in yellow), inside which crowders are excluded. Overlapping of depletion layers will increase the entropy of crowders. This is the origin of depletion forces. If the big spheres represent operons, two possibilities arise. When two operons are brought close to each other by molecular crowding, RNAPs can be redistributed within each operon or exchanged between the two. As a result, they can be viewed as soft spheres. Hard- and soft-sphere monomers are shown in (i) and (ii), respectively. The depletion force is stronger in (ii) and induces clustering of operons, as shown in (**A**). Also shown is the depletion attraction between a big monomer and a wall, in which a hard sphere picture is applicable. The varying strength of depletion forces is depicted by arrows with different thickness. (Fig. (A) is inspired by refs^3,4,30–32^).

How the ‘left’ and ‘right’ arms of the chromosome in parallel (Fig. 1(A)) are spatially organized was one of the main points addressed in recent experiments^17^. In particular, the two arms were seen to reside preferentially on the opposite sides near the inner cell membrane (Fig. 1(B)). A possibly-related observation is that membrane-protein expressing genes are localized near the cell membrane, a process that can be interpreted as maintaining the chromosome in an ‘expanded and dynamic state’^22^ (see Fig. 2(A) and relevant discussions below).

A few polymer models have been employed to gain quantitative insights into (growth-dependent) bacterial chromosome organization^5,7–10,17^. For instance, recent numerical and theoretical studies show how a linear polymer can be condensed by crowding in a confined space, similarly to what was observed with *E. coli* chromosomes^8,9^. Furthermore, a symmetric ring polymer trapped in an open cylindrical space in the absence of crowders was shown to explain the essence of observed chromosome-locus distributions in the radial direction^17^, especially separation of the two arms, as indicated by ‘near membrane’ or ‘separated arms’ in Fig. 1(B).

Despite much effort, however, how crowding influences the spatial organization of a confined polymer is far from being clear. As the chain becomes compacted by crowders, the spatial distribution of chain segments is modified in a nontrivial way^9,10^. In particular, it has been shown that crowding effects can promote chain adsorption onto the cylindrical wall^9,23^. If crowding induces depletion forces between otherwise-repelling monomers, it can induce the same kind of force between a monomer and the cylindrical wall. However, these two are “antagonizing” effects, as one hinders the other. As a result, the spatial distribution of chain segments is governed by the balance between the two effects. This has a more profound consequence on a ring polymer or a symmetrically-organized *E. coli* chromosome (see Fig. 1(A))^5,17,20,21^, since the interaction between and relative arrangement of the two arms enter into the picture. Despite its seeming simplicity, the ring polymer problem we consider here provides a rich set of organizational behavior, as evidenced below.

Using molecular dynamics simulations, here we examine how confinement and molecular crowding orchestrate in organizing a symmetrical ring polymer as a model of the *E. coli* chromosome under fast growth conditions, resembling a donut. Our results suggest that in a wide parameter space of biological relevance, the presence of crowders is essential for the emergence of (projected) two-peak distributions in the radial direction, as seen in recent experiments^17^. Under the right condition, the depletion force between each arm and the cylindrical wall dominates the radial distribution of the arms. This leads to a bimodal distribution of monomers in the radial direction, similar to what was seen with the *E. coli* chromosome^17^.

The significance of biomolecular crowding in chromosome-arm separation has yet to be tested experimentally. A related point is that chromosome loci interact with the cell membrane, for instance, through the insertion of membrane proteins in the membrane or simply ‘transertion’^17,22,24^ as illustrated in Fig. 2(A) (see ref.^25^ for a recent review). In our coarse-grained approach, this effect can be absorbed into a tuning parameter that describes the interaction of monomers with the confining cylindrical wall. In recent experiments^8^, chain adsorption was chemically discouraged; this effect was mimicked by adjusting the parameter^9^. Indeed, we find that this parameter controls the degree of chromosome-arm separation. Direct comparison between simulation and experimental data is, however, hindered by the lack of quantitative data as well as by the complexity of the chromosome associated with various proteins including RNA polymerases^3,26,27^ (see Fig. 2(A)). It is beyond the scope of this work to further clarify what factors contribute toward this parameter and to present a biological basis of it. Here, we content ourselves by mapping out possible scenarios for chromosome-arm distributions, depending on the choice of this parameter. As discussed in the last section “Discussions and Conclusions”, it is our view that the effects of crowding are favorably implicated in transcription^3,10^ and in the interaction of chromosome loci with the cell membrane.

Crowding is not the sole cause of chromosome compaction or organization. Other factors such as cationic species or proteins and supercoiling also contribute to shaping the chromosome^28^. While we do not attempt clarify the relative significance of different effects, we wish to point out that crowding effects are strong enough to condense *E. coli* chromosomes^8^. On the other hand, these additional factors are not sufficient for chromosome compaction^7,28^. In our modelling, any residual effects can be subsumed into polymer parameters similarly to our earlier attempt^5^ (also see a recent review ref.^29^). Here, we focus our effort on examining crowding effects, which we believe have remained under-explored. Our studies here suggest that crowding is not only responsible for overall compaction of bacterial chromosomes but also governs their local organization and arm distributions.

## Simulation Methods

In our simulations, we extend the simulation procedure employed for a linear chain in a cylindrical space^9^ to the case of a corresponding ring polymer. The polymer chain is a string of “simple” spherical beads or monomers; crowders are similarly modelled as simple spheres. The cylindrical wall is assumed to be formed by spherical beads, which are of the same kind as monomers. On the other hand, the solvent (water) is treated as a continuum. In principle, the quality of the solvent can be taken into account implicitly via simulation parameters. Our simulation setting represents an ‘athermal’ solvent^33^ (see refs^34,35^ for potential effects of solvent molecules).

Let *r* be the center-to-center distance between two particles (e.g., monomer-monomer, monomer-crowder, crowder-crowder pairs, …). They interact with each other through the fully-repulsive Weeks-Chandler-Anderson (WCA) potential given by ref.^36^

1 $${U}_{{\rm{W}}{\rm{C}}{\rm{A}}}(r)=\{\begin{array}{cc}4{\varepsilon }_{ij}[{(\frac{{\sigma }_{ij}}{r})}^{12}-{(\frac{{\sigma }_{ij}}{r})}^{6}+\frac{1}{4}], & \,\,r\,{ < 2}^{1/6}{\sigma }_{ij}\,\\ 0, & \,{\rm{o}}{\rm{t}}{\rm{h}}{\rm{e}}{\rm{r}}{\rm{w}}{\rm{i}}{\rm{s}}{\rm{e}}\,\end{array}.$$

Here the subscripts *i*, *j* = 1, 2, 3 are used to refer to monomers, crowders, and wall-forming particles, respectively: *σ*_11_ = *σ* = *a* (monomer size), *σ*_22_ = *a*_*c*_ (crowder size), and *σ*_12_ = *σ*_21_ = (*σ*_1_ + *σ*_2_)/2, ..., and *σ*_33_ = *σ*. Finally, *ε*_*ij*_ measures the strength of *U*_WCA_ for various interaction pairs; for instance, *ε*_11_ = *ε* represents monomer-monomer interactions and *ε*_13_ corresponds to monomer-wall interactions.

The two consecutive monomers are connected via the finitely-extensible non-linear elastic (FENE) potential: ${U}_{{\rm{FENE}}}(r)=-\frac{1}{2}k{{r}_{0}}^{2}\,\mathrm{ln}[1-{(r/{r}_{0})}^{2}]\,$, where *k* = 30.0 *ε*/*σ* ^2^ and *r*_0_ = 1.5 *σ* ^37,38^.

In addition to these interactions, any bead in our simulation, a monomer or a crowder, is subject to viscous friction and random forces, both arising from the collisions with solvent molecules. If *γ* is the viscous friction constant and **v**_*i*_ the velocity of bead *i*, the viscous force is given by −*γ* **v**_*i*_. Let *k*_B_ be the Boltzmann constant and *T* the temperature. The random force **W**_*i*_ is assumed to satisfy the relation: 〈**W**_*i*_(*t*) · **W**_*j*_(*t*′)〉 = 6*γk*_B_ *Tm*_*i*_ *δ*_*ij*_ *δ*(*t* − *t*′), where *m*_*i*_ is the mass of the bead. [Note that in this description, for simplicity, the subscript *i* is used collectively to distinguish between different bead types; *i* = 1, for instance, actually refers to a certain monomer, say the *k* th monomer (*k* = 1, 2, ..., *N*)].

The equation of motion is integrated using the velocity-Verlet algorithm^39^ with a time step 0.002*τ*_0_, where ${\tau }_{0}=\sigma \sqrt{m/\varepsilon }$ (*m* = *m*_1_ is the monomer-bead mass). We keep the system at a constant temperature given by *T* = *ε*/*k*_B_ via a Langevin thermostat^38^ (with the choice $\gamma =0.1{\tau }_{0}^{-1}$, which will not influence equilibrium quantities).

A periodic boundary condition is imposed in the longitudinal direction of the cylinder. The length of a confining cylinder is chosen to be three times the equilibrium chain size in the absence of crowders. This is to avoid possible unrealistic interactions between the polymer chain and its periodic replicas.

Initially, the polymer is organized in a helical shape but crowders are distributed randomly. After chain equilibration, we run our simulations for 5 × 10^7^ time steps and obtain data every 1000 steps. To obtain reliable chain statistics, we carried out 16 independent simulations with different initial conditions and obtained ensemble averages over all these simulations.

In our actual simulations, the simulation package LAMMPS is used^40^. Our choice of *T* above is equivalent to setting *T* = 300 K; our simulations are performed at this (room) temperature.

## Results

**Figure 3:**
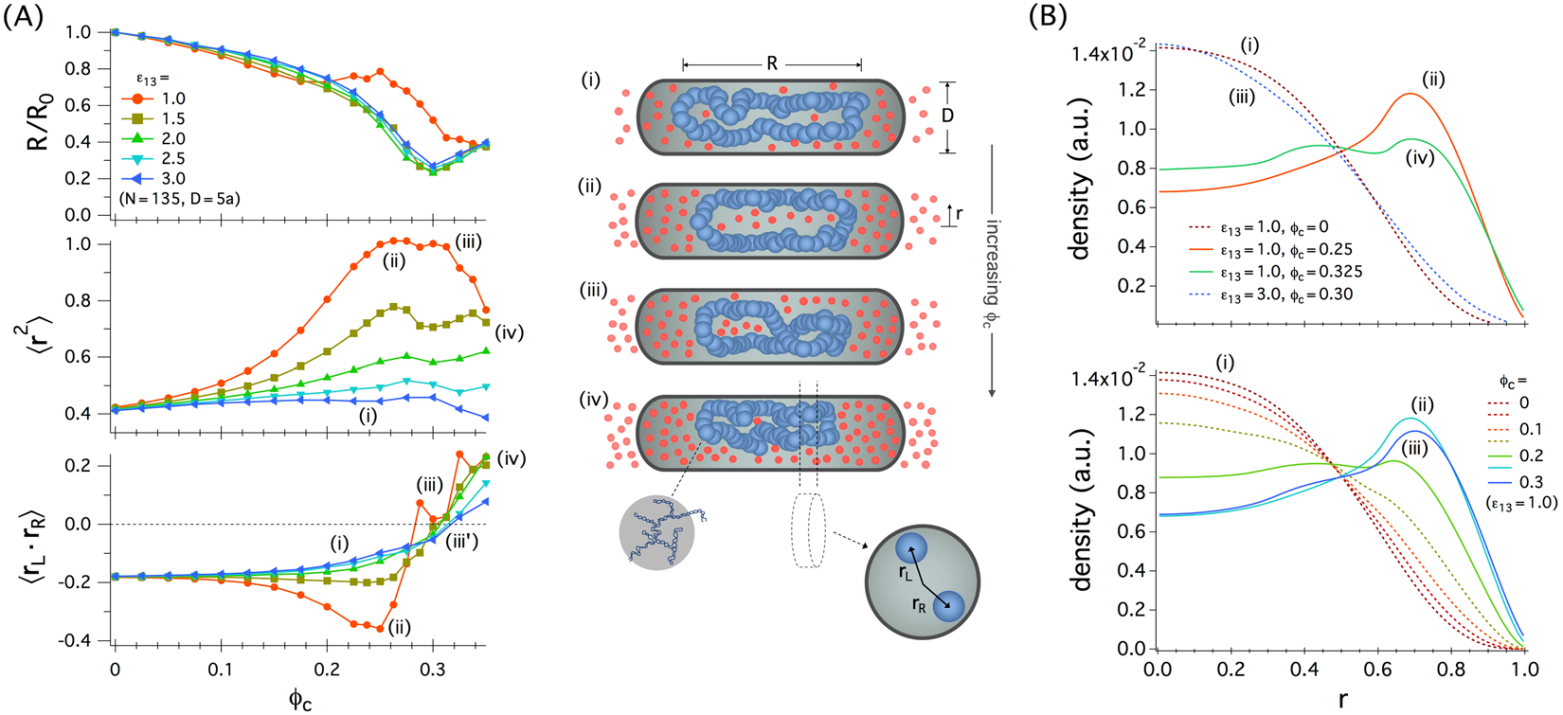
Spatial organization of a ring polymer by crowding in a confined space. (**A**) (Upper) In all these cases, the chain size *R* decreases, as the volume fraction of crowders *ϕ*_*c*_ increases. The curve for *ε*_13_ = 1.0, however, has a hump; this is a signature of a reentrant-like transition^9^. (Middle) The middle graph shows the normalized mean-squared radial position of monomers: 〈**r** ^2^〉. As the chain is adsorbed onto the cylindrical wall, this quantity approaches 1, as is the case for *ε*_13_ = 1. This supports our interpretation of a reentrant-like transition. In contrast, adsorption does not occur for *ε*_13_ = 3. (Bottom) The bottom graph displays the degree of arm separation. For small *ϕ*_*c*_, 〈**r**_L_ · **r**_R_〉 < 0 for all cases shown, as expected for the illustration (i) in the middle column. Compared to other cases, 〈**r**_L_ · **r**_R_〉 for *ε*_13_ = 1 becomes more negative with increasing *ϕ*_*c*_, as long as ${\varphi }_{c}\lesssim 0.25$. This is aligned with the finding that crowding induces chain adsorption, separating the opposing strands apart, as described by the illustration (ii). For *ε*_13_ = 1 and for a special *ϕ*_*c*_ value in the range $0.25\lesssim {\varphi }_{c}\lesssim 0.3$, 〈**r**_L_ · **r**_R_〉 ≈ 0 but 〈**r** ^2^〉 ≈ 1: the two arms are “intermingled” in the angular position near the wall, as described by (iii); in (iii′), the ring polymer is more centrally organized. As *ϕ*_*c*_ continues to increase, the two opposing strands collapse onto each other. This results in 〈**r**_L_ · **r**_R_〉 > 0 for *ϕ*_*c*_ ≈ 0.35, as illustrated in (iv). (**B**) Both the upper and lower graphs show the projected monomer density. (Upper) For the *ϕ*_*c*_ values used, the difference between *ε*_13_ = 1 and *ε*_13_ = 3 is qualitative. If the former case is characterized by the emergence of an M-shaped or bimodal distribution, the latter lacks this feature. (Lower) This shows how the distribution for *ε*_13_ = 1.0 is modified as *ϕ*_*c*_ changes. The emergence of a bimodal distribution is a result of the depletion forces that tend to bring the opposing arms away from each other toward the wall.

We employ a ring polymer trapped in a cylindrical space as a coarse-grained chromosome and examine how crowding influences the organization of ring arms (shown in green and orange in Fig. 1(A)). Each monomer may be viewed as the structural unit of the chromosome, consisting of supercoiled DNA and DNA-associated proteins (see refs^8,29^ and references in therein), as illustrated at the bottom of the middle column in Fig. 3 as well as in Fig. 1(A). This was inspired by our earlier observation that when constructed with caution, this model or its variations have been useful for understanding the large scale organization of chromosomes^5,8,29^. At length scales larger than the size of the structural unit (~100 nm), the notion of DNA persistence length becomes less meaningful; because of supercoiling and compaction, each unit contains many persistence lengths. For more microscopic studies (e.g., DNA supercoiling, knotting, and looping), however, the DNA structure has to be explicitly considered (see for instance ref.^41^).

We primarily choose the cylinder diameter *D* = 5*a*, the number of monomers *N* = 135, and the crowder size *a*_*c*_ = 0.3*a*; we also use a sizeable range of *ϕ*_*c*_ (the volume fraction of crowders) and several choices of *ε*_13_ (the interaction parameter for a monomer and the cylindrical wall) given in units of *ε* = *ε*_11_ (the interaction parameter for monomers): *ϕ*_*c*_ = {0, …, 0.35} and *ε*_13_ = {1, 1.5, …, 3.0}. The temperature is set to *T* = 300K. The equilibrium chain size in the absence of crowders is thus comparable to that of a linear chain for *N* = 80: *R*_0_ = *R*(*ϕ*_*c*_ = 0) ≈ 26*a*.

### Ring-arm distribution in the presence of crowders

In the absence of crowding, the effect of ring topology under cylindrical confinement is “quantitative”. A ring polymer can be viewed as a “parallel connection” of two arms (or sub-chains), with each arm trapped in a narrower imaginary tube^42^. This analogy is not generally true in the presence of crowders, because of depletion forces between the cylindrical wall and the arm. Indeed, crowding can induce attractive depletion forces not only between two monomers^15,16,43^ but also between a monomer and the cylindrical wall^9,23^ as illustrated in Fig. 2(B) (see the hard-sphere case (i)). This can be understood in terms of ‘depletion layers’ (in yellow in Fig. 2(B))^15,16,43^. Overlapping of depletion layers is favored by the entropy of crowders. This is the physical origin of depletion forces, promoting chain compaction or adsorption; they reflect the geometry of the confining space through the spatial distribution of crowders, which follows the shape of the cylinder wall. As a result, the interplay between compaction and adsorption will govern the spatial organization of the confined chain. For the ring polymer we consider here, the ring topology will enter into the picture: the depletion force will influence the relative positioning of its two arms.

For this reason, we note that three quantities are needed to fully characterize a ring polymer, as shown in Fig. 3: (i) the longitudinal chain size *R* or the length of an imaginary tube enclosing the ring, (ii) the mean-squared radial position of monomers 〈**r** ^2^〉 (not to be confused with *r* in Eq. 1), and (iii) the angular correlation between the two monomers on the opposing arms or subchains of the ring arranged in parallel 〈**r**_L_ · **r**_R_〉. Here, **r**_L_ and **r**_R_ are the positions of monomers on the left and right arm, respectively. The ensemble average, denoted as ⟨ . . . ⟩, is taken over all monomers as well as over different realizations. As a result, it does not depend on the contour position of monomers.

We have measured and plotted these quantities as a function of *ϕ*_*c*_ in Fig. 3(A). First, the upper panel shows a graph of rescaled chain sizes *R*/*R*_0_ vs. *ϕ*_*c*_ for various choices of *ε*_13_, where *R*_0_ = *R*(*ϕ*_*c*_ = 0). (The rescaled quantity *R*/*R*_0_ is *D*-independent^9^). In all these cases, the confined chain shrinks in size, as *ϕ*_*c*_ increases (see refs^9,33^ for the physical origin of a non-monotonic tail for ${\varphi }_{c}\gtrsim 0.3$). In contrast to others, the curve for *ε*_13_ = 1.0 has a bump at *ϕ*_*c*_ ≈ 0.24. This is a signature of a reentrant-like transition as seen with a linear chain^9^. It can be understood in terms of the competition between chain compaction and adsorption^9^ both induced by crowding, as illustrated in Fig. 2(B); the depletion force between a sphere and a surface is generally stronger than that between two spheres^3^. Compaction and adsorption are, however, competing phenomena, as one hinders the other; in order for the chain to allow more contacts with the cylindrical wall, it has to stretch in the longitudinal direction, possibly at the expense of depletion interactions among monomers. As a result, initially, crowding condenses the chain longitudinally such that the (normalized) radial distribution remains roughly unchanged. This is a distinguishing feature of anisotropic confinement; it would be easier for the crowders to compress the already-radially compressed chain in the longitudinal direction (unless the crowders reside preferably near the cylinder wall, as is the case for a stiff chain like DNA^2,29^).

For small *ε*_13_ (i.e., *ε*_13_ = 1 represented by the red curve), the competition becomes favorable to adsorption, as *ϕ*_*c*_ increases up to ≈0.25; the crowding effect switches to promote chain adsorption, elongating the chain along the cylinder, which would otherwise remain condensed. This is a natural consequence of stronger depletion forces with a surface^3^ (see Fig. 2(B)). It is responsible for a reentrant-like transition, as indicated by the red curve. As *ϕ*_*c*_ increases further beyond *ϕ*_*c*_ ≈ 0.25, the balance is tilt toward chain compaction, i.e., crowding effects are strong enough to condense the chain in the longitudinal direction.

The middle panel in Fig. 3(A) shows the mean-squared radial position of monomers 〈**r** ^2^〉; here and below, **r** is the radial position vector rescaled by *D*/2 (so are **r**_L_ and **r**_R_). This quantity gives an average sense of where the monomers are in the radial direction. For a uniform distribution, 〈**r** ^2^〉 = 0.5. As with a corresponding linear chain, for *ϕ*_*c*_ = 0, the chain slightly prefers to be in the middle rather than on the cylindrical wall, i.e., 〈**r** ^2^〉 ≈ 0.4. As the chain is adsorbed onto the cylindrical wall, this quantity approaches 1. Indeed, this is the case for *ε*_13_ = 1 under the right condition ($0.25\lesssim {\varphi }_{c}\lesssim 0.3$), as illustrated in the middle panel (see (ii)). This is well aligned with our interpretation of a reentrant-like transition (the red curve in the upper panel). Beyond *ϕ*_*c*_ ≈ 0.3, 〈**r** ^2^〉 decreases with increasing *ϕ*_*c*_; this is a consequence of the two arms collapsing onto each other (see below). In contrast, adsorption does not occur for *ε*_13_ = 3. In this case, 〈**r** ^2^〉 is roughly independent of *ϕ*_*c*_, similarly to what was seen with a linear chain under similar conditions^9^.

The last rescaled quantity in Fig. 3(A) (bottom panel), 〈**r**_L_ · **r**_R_〉, primarily measures the angular correlation between two monomers on the opposing arms or subchains. (This quantity is averaged not only over different realizations of **r**_L_ or **r**_R_ but also over monomers on each arm.) If 〈**r**_L_ · **r**_R_〉 ≈ 1, the two monomers are at the same “clock” or angular position and reside near the cylinder wall; if 〈**r**_L_ · **r**_R_〉 ≈ −1, they tend to be on the opposite sides near the wall (see the illustration (ii) on the right). As shown in the figure, for small *ϕ*_*c*_, 〈**r**_L_ · **r**_R_〉 < 0 for all cases shown; except for *ε*_13_ = 1, this quality is roughly insensitive to *ϕ*_*c*_ as long as ${\varphi }_{c}\lesssim 0.25$. This is a direct consequence of the repulsive excluded-volume interaction between monomers on the opposite sides in the radial direction. But for *ε*_13_ = 1, 〈**r**_L_ · **r**_R_〉 (<0) becomes more negative with increasing *ϕ*_*c*_, as long as ${\varphi }_{c}\lesssim 0.25$. This is correlated with the finding that crowding induces chain adsorption, separating the opposing strands further.

The pronounced non-monotonic dependence of 〈**r**_L_ · **r**_R_〉 on *ϕ*_*c*_ is unique to the case *ε*_13_ = 1. In this case, the arm separation is most noticeable when *ϕ*_*c*_ ≈ 0.25, as best described by the arm distribution labelled as (ii) in Fig. 3 (see the illustration in the middle column). This is somewhat analogous to the ‘separated-arm’ distribution in Fig. 1(B), in which the two arms are on the opposite clock or angular positions. For *ε*_13_ = 1 and for a special *ϕ*_*c*_ value in the range $0.25\lesssim {\varphi }_{c}\lesssim 0.3$, 〈**r**_L_ · **r**_R_〉 ≈ 0 but 〈**r** ^2^〉 ≈ 1 (see (iii)). This is an indication of the two arms “intermingled” near the wall in the sense that they are randomly distributed in the angular position. The resulting arm distribution seems to resemble the ‘near-membrane’ distribution in Fig. 1(B). The main difference between ‘near membrane’ and ‘separated arms’ in Fig. 3 is that the angular correlation 〈**r**_L_ · **r**_R_〉 is small in the former but large negative in the latter case. Making a more quantitative comparison between Figs 1(B) and 3 is not so obvious; it is not clear how the distributions in Fig. 1(B) can be translated into the angular correlation 〈**r**_L_ · **r**_R_〉. For a larger *ε*_13_ value (e.g., blue and cyan curves), however, 〈**r**_L_ · **r**_R_〉 ≈ 0 as for *ε*_13_ = 1.0 but 〈**r** ^2^〉 ≈ 0.4, in the same range of *ϕ*_*c*_. The chain is somewhat centrally organized and the resulting organization is referred to as (iii′).

In all cases shown in the bottom graph in Fig. 3(A), however, as *ϕ*_*c*_ continues to increase, 〈**r**_L_ · **r**_R_〉 increases monotonically or non-monotonically to a positive value for *ϕ*_*c*_ ≈ 0.35. This means that the two opposing strands eventually collapse onto each other, as described by the illustration (iv) in the middle column. In this case, the distinction between different cases becomes less significant. The arm distribution labelled as (iii) appears to combine both features of (ii) and (iv).

Inspired by recent experiments with *E. coli* chromosomes, in which the radial organization of the the chromosome was examined^17^, we have measured the radial density of monomers *ρ*(*r* = |**r**|) projected onto a plane that contains the long symmetry axis, averaged over all possible chain conformations (as well as over all monomers). Our results for *ρ*(*r*) are displayed in Fig. 3(B); we have chosen the same parameters, i.e., *N* = 135 and *D* = 5*a*, as in Fig. 3(A). While the results in Fig. 3(B) are well aligned with some of those in Fig. 3(A), the difference between *ε*_13_ = 1.0 and *ε*_13_ = 3.0 is captured better and appears to be more qualitative in Fig. 3(B). A salient feature of the case *ε*_13_ = 1.0 is the appearance of a two-peak or bimodal monomer distribution in the radial direction. This observation is correlated with the corresponding curve (*ε*_13_ = 1.0) in the bottom graph in Fig. 3(A), showing a large negative angular correlation for *ϕ*_*c*_ ≈ 0.25: the two opposing arms are pushed away from each other toward the cylinder wall.

The lower panel in Fig. 3(B) displays the density distribution for the case *ε*_13_ = 1.0 and for various choices of *ϕ*_*c*_. In particular, it shows how a bimodal shape emerges, when *ϕ*_*c*_ = 0.25 or 0.3. Taken together with Fig. 3(A), the emergence of a bimodal distribution can be attributed to the depletion forces that bring the opposing arms away from each other toward the cylindrical wall.

The results in Fig. 3(B) suggest that the presence of crowders (*ϕ*_*c*_ ≈ 0.25–0.3) is required for this bimodal behavior. This offers a quantitative basis of what was observed with symmetrically-organized *E. coli* chromosomes: the chromosome loci somewhere in the middle also show a bimodal density distribution^17^. Indeed, cells are crowded with various molecules including water-soluble cytoplasmic proteins^44,45^. While our coarse-grained system differs from the biological counterpart in detail, we expect that the essence of how these cellular crowders in *E. coli* would organize its chromosome is qualitatively-correctly captured in the results in Fig. 3(A) and (B).

**Figure 4:**
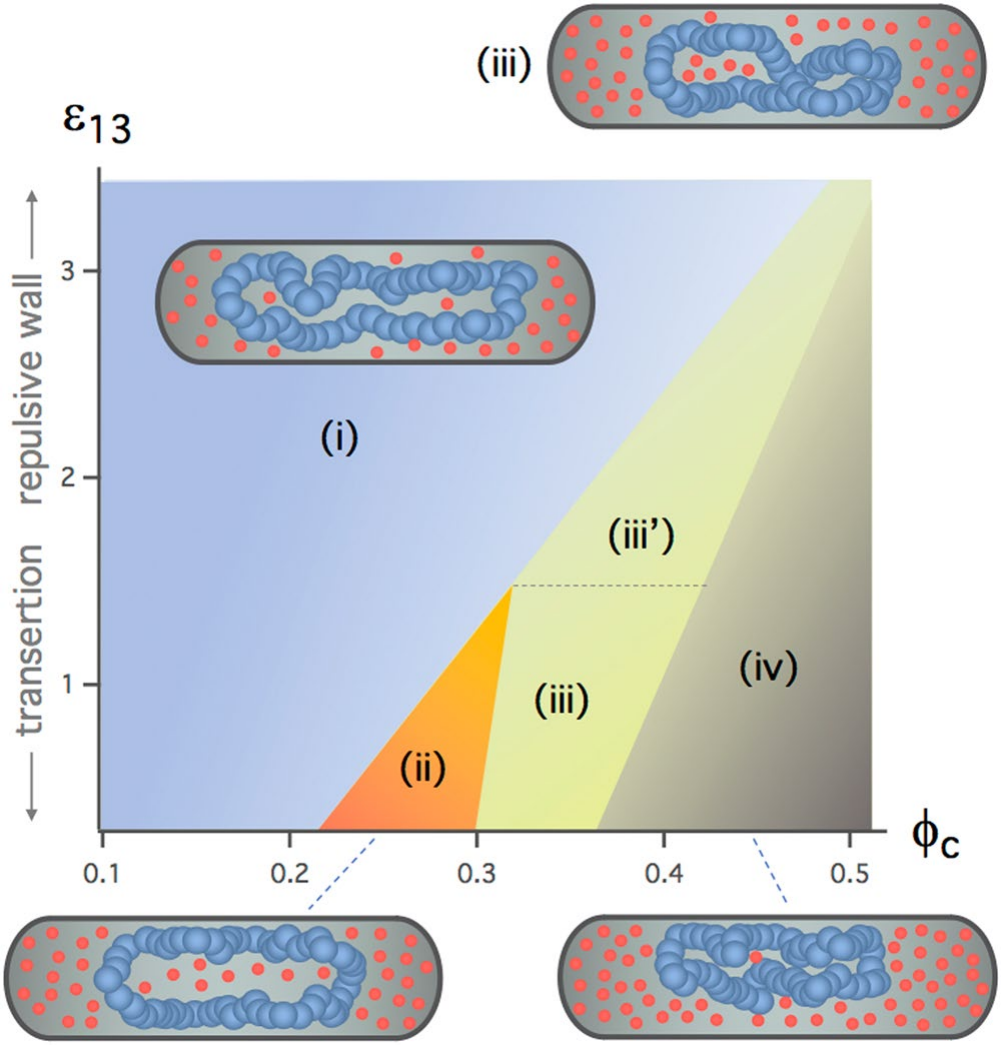
Ring-arm organization diagram. The diagram shows various regions in a *ϕ*_*c*_ − *ε*_13_ space, in which the ring polymer assumes the arm distributions labelled as (i), … (iv) in Fig. 3. First note that region (i) is realized for a wider *ϕ*_*c*_ range for a larger *ε*_13_ value. This sets the boundary of this region. On the other hand, the emergence of region (ii) requires small *ε*_13_ and some intermediate *ϕ*_*c*_-range: the smaller *ε*_13_ is, the larger *ϕ*_*c*_ range is, as indicated by the shape of this region. Region (iv) can be entered more easily if *ε*_13_ is smaller. Between (i) and (iv) or between (ii) and (iv) is the regime (iii) (or (iii)). Note that the boundary between neighboring regions is model-dependent; in particular, the *ϕ*_*c*_ range required for a given region is specific to our choice of *a*_*c*_ (=0.3*a*).

The results in Fig. 3 suggest that ring-arm distributions depend on *ε*_13_ and *ϕ*_*c*_. The diagram in Fig. 4 summaries the resulting arm distributions in a *ϕ*_*c*_ − *ε*_13_ space. It shows schematically various regions, in which the ring polymer takes on the arm organization labelled as (i), …, (iv) in Fig. 3. First note that region (i) is realized for a wider *ϕ*_*c*_ range for a larger *ε*_13_ value. This sets the boundary of this region. On the other hand, the emergence of the next region (ii) requires small *ε*_13_ values and some intermediate *ϕ*_*c*_-range: the smaller *ε*_13_ is, the larger *ϕ*_*c*_ range is, as indicated by the shape of this region. The largest-*ϕ*_*c*_ region (iv) can be entered more easily if *ε*_13_ is smaller. Between (i) and (iv) or between (ii) and (iv), there lies region (iii) (or (iii′)).

Note that the boundary between different regions is a smooth crossover. Also, it depends somewhat on how they are constructed. For instance, in the region (iv), the angular correlation between the two arms 〈**r**_L_ · **r**_R_〉 is positive. If 〈**r**_L_ · **r**_R_〉 is set to a larger value, this region will be narrower. If so, the blue curve in Fig. 3(A) will not easily fall in this region. This is reflected in the diagram in Fig. 4. As discussed in detail^10,33^, the “quality” of crowders depends crowder size *a*_*c*_. Assuming that *a* > *a*_*c*_, for smaller crowders, a smaller value of *ϕ*_*c*_ is needed for the same level of compaction. This implies that the required *ϕ*_*c*_ range for a given region depends on crowder sizes^10^. The numbers shown in the diagram should not be taken too literally.

### Ring-arm distribution in the absence of crowders

A coarse-grained polymer model was already used to explain how the bimodal distribution arises: a ring polymer in an open cylinder without crowders^17^. In contrast to what the results in Fig. 3 suggest, a bimodal distribution was observed in the absence of crowders. In this modelling, *D* = 3*a* was used. To focus on confinement effects and to reconcile any discrepancy, we have repeated our simulations in the absence of crowding; the main difference is that a ring polymer is now trapped in a cylindrical space of length *L*, which can be smaller than *R*_0_, as for the bacterial chromosome.

**Figure 5:**
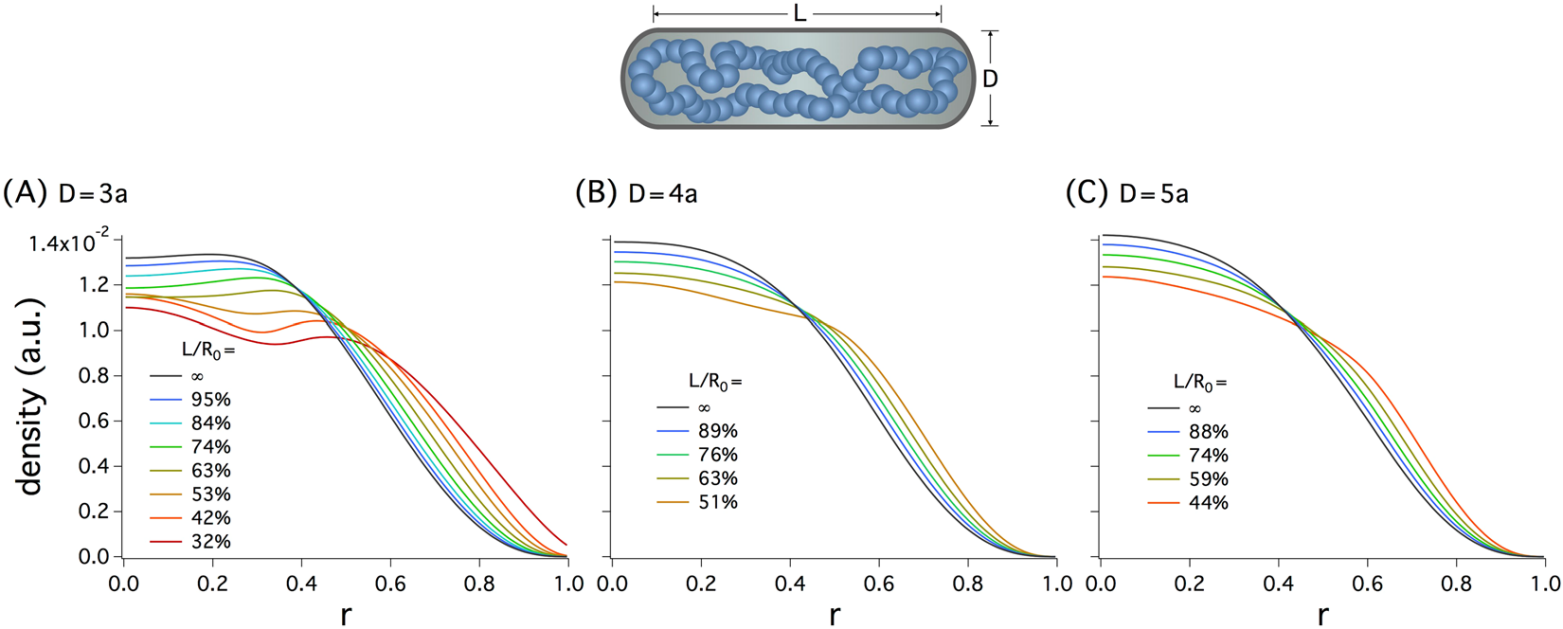
Monomer distributions in the radial direction in the absence of crowding for *N* = 135 and for various choices of *D* and *L*/*R*_0_, where *L* is the cylinder length and *R*_0_ the relaxed chain length: (**A**) *D* = 3*a*, (**B**) *D* = 4*a*, and (**C**) *D* = 5*a*. Among them, only the case *D* = 3*a* shows a bimodal distribution, especially when $L/{R}_{0}\gtrsim \mathrm{60 \% }$. For *L*/*R*_0 _≤ 53%, the monomer density has a maximum at *r* = 0 and develops a bump toward the cylindrical wall; similar to what was seen earlier^46,47^; at high monomer densities, monomers can get layered near the wall. For other values of *D* in (**B**) and (**C**), the monomer density in the radial direction has a single peak at *r* = 0 for all choices of *L*/*R*_0_ used, possibly except for *L*/*R*_0_ = 51% in (**B**). Together with those in Fig. 3, the results in this figure suggest that molecular crowding is required for the bimodal monomer distributions of a ring polymer in a cell-like confined space.

Figure 5 displays our results for projected monomer distributions in the radial direction for various combinations of *D* and *L*/*R*_0_: (A) *D* = 3*a*, (B) *D* = 4*a*, and (C) *D* = 5*a*. Among them, only the case *D* = 3*a* shows a bimodal-like distribution, especially when $L/{R}_{0}\gtrsim \mathrm{60 \% }$; even for the open cylinder case (*L*/*R*_0_ = ∞) described by the curve in black, however, the distribution is rather broad and appears to be somewhat distinct from the bimodal distributions in Fig. 3. For *L*/*R*_0_ ≤ 53%, the monomer density has a maximum at *r* = 0 and develops a “bump” near the cylindrical wall; at high monomer densities, monomers can get wall-layered, creating a bump^46,47^. For other values of *D* in (B) and (C), the monomer density in the radial direction has a single peak at *r* = 0 for all choices of *L*/*R*_0_ used, possibly except for the case *L*/*R*_0_ = 51% in (B). Together with those in Fig. 3, the results in this figure suggest that molecular crowding is required for the separation of two arms of a ring polymer in a cell-like confined space, as marked by a bimodal monomer distribution. (In Fig. 3, the ring polymer was compressed longitudinally by molecular crowding, whereas in Fig. 5, it was compressed by the cylinder caps at both ends of the cylinder.) Otherwise, monomers tend to be centrally positioned or broadly distributed in the radial direction.

In summary, our work highlights the significance of molecular crowding in organizing a ring polymer in a cell-mimicking cylindrical space. Compared to the corresponding linear-chain or unconfined case^9,10,33^ the confined ring polymer shows much richer organizational properties. In a parameter space of biological relevance (e.g., *ϕ*_*c*_ ≈ 0.2–0.3), molecular crowding not only compresses the chain longitudinally but also can separate the two arms, in the way seen with *E. coli* chromosomes at fast growth rates^17^. Under different conditions (e.g., enhanced repulsions between monomers or the cylindrical wall or different ϕ_*c*_ values), the ring polymer is centrally condensed, marked by unimodal monomer distributions; or it is adsorbed onto the cylindrical wall with the two arms laterally collapsed onto each other.

## Discussions and Conclusions

In conclusion, our results show how molecular crowding can modify qualitatively the spatial distribution of a ring polymer in a confined space. With the right parameter choices, the ring polymer shows chromosome-like organization in *E. coli* (e.g., bimodal locus distributions in the radial direction). The crowding effects induced by cytoplasmic crowders not only enable the chromosome to occupy a subspace in the cell but also assist it in assuming a desired structure for biological functions, e.g., chromosome-arm separation as for membrane-protein expression^22^ and the clustering of ribosomal RNA operons^3,10^ (see Fig. 2).

In general, how molecular crowding influences the spatial organization of a long chain molecule under cylindrical confinement depends on various parameters: chain persistence length and thickness as well as crowder size and type^2,10,29^. When the chromosome is coarse-grained into a string of monomeric units, each unit, containing many persistence lengths, is much more spherical than what the naked DNA might naively suggest^29^. In some studies including this work, it was chosen to be a structural unit or a topological domain^8,9,17,29^.

It is worth commenting on biological complexities our polymer approach leaves out, clarifying its limitations. Most obviously, because of DNA replication, the bacterial chromosome is dynamic and topologically complex^17^; also the DNA packing level is variable. As a result, the chromosome can only locally equilibrate. Also the process of chromosome segregation (e.g., *oriC* driven toward the pole) can modify locus distributions^17,48,49^. It is gratifying to note that chromosome arms remain well-separated in the radial cell axis, largely independent of replication states^17^. This is consistent with our view that the distributions of chromosome loci are governed by simple physical principles; it may justify our equilibrium picture of chromosome organization based on a coarse-grained model of chromosomes (see ref^17^ for an earlier attempt).

Furthermore, the cellular crowders are poly-disperse in size (see Fig. 2(A)). In particular, ribosomes are appreciably larger (≈20 nm in diameter) than average crowders (≈5 nm), as schematically shown in Fig. 2(A) ^50^. There is much evidence that big crowders do not mix well with small ones, which in turn influences the spatial organization of chain molecules they surround^10^. Together with the results in Fig. 3 and those reported earlier^9^ this indicates that big crowders are preferentially distributed in the pole region (see refs^30,31^ for biological details), contributing to longitudinal compression of the chromosome; it is easier for the chromosome to be compressed longitudinally than radially.

As noted earlier, chain heterogeneity appears to be a distinct feature of the biological ring polymer, i.e., the circular bacterial chromosome. The significance of this biological complexity may depend on what aspect of chromosomes one wishes to explore. For instance, the binding of RNA polymerases (RNAPs) on ribosomal RNA (rRNA) operons in fast growing *E. coli* can facilitate clustering of the operons into the so-called ‘transcription foci’^3,10,27,32^ (see Fig. 2(A) as well as refs^51,52^). Each rRNA operon, represented by a big sphere in cyan in Fig. 2(A), contains many (≈70) RNAPs. In a polymer model, the binding can be considered as enlarging the size of monomers, as illustrated in Fig. 2(A). The entropic (depletion) force is stronger between big monomers. This is responsible for the clustering of big monomers^3,10^.

The details of polymer modelling depend on the physical picture of operons, which are represented by big spheres in cyan in Fig. 2. Each RNAP is about 10nm in size^32^. If seventy RNAPs are assumed to be tightly packed, each big sphere is as large as ≈40 nm in diameter^3,10^. This may serve as a lower bound. A larger value will be reached if each RNAP is modelled as a 16 nm sphere^53^. More realistically, one can view the big sphere as a cluster of loosely packed RNAPs together with the intervening DNA and rRNAs^53^. In all these cases, crowding effects are strong enough to induce the clustering of big spheres (see also relevant discussions in ref.^10^).

There is, however, a subtle difference between the bead-spring polymer, consisting of big and small monomers, and the biological counterpart. RNAPs can be exchanged between adjacent operons^3^. As a result, two neighboring transcription units (big spheres in cyan in Fig. 2) can merge into a bigger one and thus experience a stronger depletion force than the corresponding hard spheres do^3^. Their clustering will not be easily interfered by adsorption, as indicated in Fig. 2(B). Because of the lack of relevant data, the potential effects of clustering on the radial distributions of chromosome loci are largely unknown but can be clarified.

A more directly-related observation is that genes actively-expressing membrane proteins in *E. coli* reside preferentially near the cell membrane^22^. An emerging picture is that the *E. coli* chromosome is in an ‘expanded and dynamic state’. This will not necessarily contradict the clustering of rRNA operons, which was claimed to make the chromosome more compact^32^. While membrane-protein encoding genes are distributed throughout the chromosome^22,54^ rRNA operons are mostly concentrated near *oriC* ^3,27,55^. Similarly to the way crowding benefits transcription, it is our view that it makes more favorable the interaction of chromosome loci with the (inner) cell membrane, as desired for membrane-protein expression. A combined effort between experiments and polymer modelling will be useful for further clarifying how crowding and transertion are interrelated.

Also, unclear is how molecular crowding controls the organization of a heterogeneous polymer, especially in a poly-disperse crowded medium under confinement (see refs^3,10^ for recent attempts). Both monomer and crowder sizes modify crowding effects and thus the spatial distributions of monomers and crowders^10,33^. Furthermore, the local organization of a heterogeneous polymer (e.g., clustering) occurs at the expense of chain entropy (see for instance ref.^3^); while the association of two big monomers is promoted by their depletion attraction, the intervening section of the chain will suffer from chain-entropy reduction. It thus depends on the contour position of big monomers as well as on the overall orientation of the polymer with respect to the long axis of the cylinder. Further considerations will be useful for refining our polymer-chromosome model.
